# Neonatal manifestations in COVID-19 patients at a Brazilian tertiary center

**DOI:** 10.6061/clinics/2020/e2407

**Published:** 2020-11-10

**Authors:** Glenda Priscila Neves dos Santos Beozzo, Werther Brunow de Carvalho, Vera Lucia Jornada Krebs, Maria Augusta Bento Cicaroni Gibelli, Romy Schmidt Brock Zacharias, Larissa Elizabeth Schulz Rossetto, Rossana Pulcinelli Vieira Francisco

**Affiliations:** I Departamento de Pediatria, Faculdade de Medicina (FMUSP), Universidade de Sao Paulo, Sao Paulo, SP, BR; II Departamento de Ginecologia e Obstetricia, Faculdade de Medicina (FMUSP), Universidade de Sao Paulo, Sao Paulo, SP, BR

The most common symptoms of severe acute respiratory syndrome coronavirus 2 (SARS-CoV-2) infection in children are fever and cough, which are present in approximately 59% of cases (1). Other symptoms include bloating, nausea, vomiting, diarrhea, and abdominal pain. Although the evolution is generally benign, the clinical picture of SARS-CoV-2 infection in neonates is still poorly understood (2). It is always necessary to consider that severe respiratory distress can also occur in other conditions that are inherent to pulmonary immaturity at this stage of life. Although most newborns are able to make a full recovery from SARS-CoV-2 infection, there have been reports of sepsis, severe respiratory failure, and shock (3,4).

A total of 49 newborns were born to mothers with proven SARS-CoV-2 infection between April and June 2020 in a Brazilian tertiary center. These neonates received a SARS-CoV-2 test by reverse transcriptase polymerase chain reaction (RT-PCR) using nasal and oropharynx secretions. As a result, two newborns tested positive 48 hours after birth and two positive newborns were admitted to the Unit as out-born cases, one of whose case has already been reported in a previous study (5).

**Case 1:** Case 1 was that of a baby DGL, term newborn, son of a mother who had flu-like symptoms 4 days before delivery, and who tested positive for SARS-CoV-2 by RT-PCR of nasal and oropharyngeal secretions. The mother had a vaginal delivery, with rupture of ovular membranes 2 h prior. After 24 h of life, the newborn presented with episodes of sinus bradycardia (heart rate<90 bpm) confirmed by electrocardiogram, accompanied by hypocalcemia, with a normal echocardiogram. He received calcium gluconate orally for 72 h, and the bradycardia subsequently resolved. On the 2^nd^ and 3^rd^ days of life, his oropharyngeal swab tests (RT-PCR) were positive for SARS-CoV-2. On the 6^th^ day of life, the oropharyngeal swab (RT-PCR) for SARS-CoV-2 and the serum measurement of immunoglobulin G (IgG) antibodies for SARS-CoV-2 were negative. During hospitalization, he remained afebrile in room air, and he was discharged at 8 days old.

**Case 2**: Case 2 was that of a baby ACO, preterm with a gestational age of 33 4/7 weeks, female born by cesarean delivery due to maternal clinical decompensation. During pregnancy, the mother presented with sepsis and urinary tract infection by *Escherichia coli*, severe anemia, caloric protein malnutrition, and chronic cough. The mother’s respiratory condition worsened 10 days before delivery, and she tested positive by SARS-CoV-2 RT-PCR with a nasopharyngeal swab. In the 2^nd^, 3^rd^, and 17^th^ days of the newborn female’s life, SARS-CoV-2 RT-PCR of an oropharynx swab was positive. On the 3^rd^ day of life, she presented with episodes of decreased O_2_ pulse saturation and respiratory distress and required inhaled oxygen until the 14^th^ day of life. Chest radiography performed at 5 days of life showed no pulmonary changes. The results of chest computed tomography and pulmonary ultrasound examination 17 days of life are shown in Fig. 1. The echocardiogram was normal, and the head ultrasound showed grade II intraventricular hemorrhage. At 22 days of life, he had an episode of bleeding in the stool and anemia (hemoglobin=7.6 g/dL) and required transfusion of packed red blood cells. On the same occasion, the nasopharyngeal swab RT-PCR was negative for SARS-CoV-2, while the blood IgG antibody test was positive. She remained afebrile during hospitalization and was discharged at 26 days of life.

**Case 3:** Case 3 was that of a JCR, term newborn, son of a healthy and asymptomatic mother who had a vaginal delivery. The newborn had a birth weight of 3600 g, and he was discharged from the hospital with 48 h of life. At 8 days of life, the newborn presented with a fever of 38°C with no other symptoms. Following medical evaluation, urinary tract infection was diagnosed, with *Escherichia coli* testing positive in urine culture, and he was subsequently started on IV antibiotics. In the newborn's home, the parents and grandmother had flu-like symptoms, while during hospitalization, the newborn had nasal congestion and a runny nose. SARS-CoV-2 by RT-PCR in nasopharyngeal swabs was positive, and chest radiography showed no abnormalities. Chest ultrasound at 18 days of life (6 days of disease progression) showed bilateral coalescent B lines on both sides of the chest (P3 classification). Chest tomography on the 7th day of the disease (19 days of life) is shown in Fig. 2. He did not require supplemental oxygen over the course of his hospitalization; he was afebrile and had an O_2_ pulse saturation above 94%. He was discharged from the hospital after treatment for the urinary tract infection at 23 days of life.

The clinical findings and laboratory tests of the three patients are shown in Tables 1 and 2.

In patients 1 and 2, the mothers had respiratory symptoms before delivery, with a positive SARS-CoV-2 test by RT-PCR in nasal and oropharyngeal secretions on the day of delivery and 10 days before delivery, respectively. The delivery took place at the institution, and the newborn was immediately isolated from the mother. Although it is possible that vertical transmission occurred in cases 1 and 2, no examinations of the placenta and amniotic fluid were performed. The parents and grandmother of case 3 had flu-like symptoms; thus, in this neonate, whose age at diagnosis of SARS-CoV-2 infection was 11 days, the most likely source of infection was postnatal contamination by contact with infected persons at home.

Premature birth has been reported more frequently in pregnant women with SARS-CoV-2 infection, which can occur in up to 47% of cases (6).

The clinical signs and symptoms of SARS-CoV-2 infection in neonates appear to be different from those in older children and adults. The presence of fever, one of the main symptoms reported in older children and adults with SARS-CoV-2 infection (1,2), was only observed in case 3, where it was transient. Furthermore, case 3 had a concomitant urinary tract infection with *Escherichia coli*, which may have been the source of the fever. In a series of seven infants aged 60 days who presented with fever, with no other symptoms, and confirmed diagnosis of SARS-CoV-2 infection (7), two infants was concomitant diagnosed *Escherichia coli* urinary tract infection. We consider the concomitant occurrence of bacterial infection of the urinary tract and SARS-CoV-2 infection to be relevant in the presence of neonatal febrile conditions. In our opinion, research on SARS-CoV-2 infection should be included in the approach to fever without localized signs in neonates.

One fact that should be highlighted is the dissociation between respiratory symptoms and the severity of the tomography and lung ultrasound images. Despite the injuries observed, the neonates remained asymptomatic or slightly symptomatic, and did not require ventilatory support.

Likewise, in most patients, the significant increase in D-dimer levels was not accompanied by clinical symptoms related to coagulopathy. Only the preterm newborn (case 2) experienced an episode of bleeding in feces, grade II intracranial hemorrhage, and anemia. It is possible that the prematurity itself contributed to these clinical symptoms. Although changes in D-dimer levels are associated with poor prognosis in adults (8,9), the other two patients who underwent coagulation tests (cases 1 and 3) did not present with complications related to blood coagulation abnormalities.

It is important to compare the positive serology of case 2, whose mother had a prolonged history of coughing before delivery, to the negative serological result of case 1, whose mother had symptoms for only 4 days. These findings suggest that the presence of antibodies in case 2 occurred due to passive transfer of maternal antibodies.

McLaren et al. (7) reported that there was no need for mechanical ventilation or admission to the neonatal intensive care unit (NICU) in infants up to 60 days of life who were had a positive SARS-CoV-2 test result. The authors reported that these children generally show a favorable outcome. However, it might not be possible to make this statement in cases detected in the newborn period, as it represents a more vulnerable population with unknown outcomes.

In our series, all neonates progressed well and were discharged, in agreement with the findings of Lu and Shi, who reported three newborns infected at home, with tachypnea, vomiting, cough, and fever, all of which evolved without complications (10). However, some authors have described serious complications and death. Coronado et al. (11) reported a 3-week old late preterm newborn, who presented with fever and tachypnea, with pneumonia present on the radiological image. The SARS-CoV-2 test was positive, and the patient presented with septic shock and pneumothorax.

Our cases were admitted to the NICU for monitoring despite being clinically well, because of clinical signs, abnormal clotting, anemia, and chest tomography with lung parenchyma lesions. In addition, newborns can easily become seriously ill and mortality is high, with infections being one of the main causes of death (12). Furthermore, considering the small number of cases published in the literature so far, the performance of complementary tests and monitoring of newborns with SARS-CoV-2 in the NICU should always be considered.

Although there were 49 newborns from mothers with confirmed SARS-CoV-2 infection, only two newborns had positive SARS-CoV-2 test results, with case 3 referred from another health care center. We agree with Zhu et al. (13) in that we consider any clinical finding to be important, even in the presence of a negative SARS-CoV-2 swab test in the newborn of a mother with COVID-19, since false-negative results are possible (13,14).

We conclude that the clinical presentation of SARS-CoV-2 infection was variable in the three neonates reported above. Fever only occurred in a newborn who became infected at home, and the disease progression of the patient was favorable. However, the dissociation between the symptoms, the findings on lung imaging tests, and the D-dimer levels in these three cases suggest the potential risk of serious complications.

## Figures and Tables

**Figure 1:**
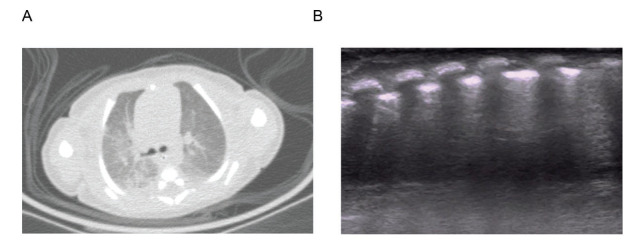
Baby ACO pulmonary imaging exam (case 2). A. Chest computed tomography: Opacities and atelectasis band in the right upper lobe. Slightly diffuse increase in attenuation of the lung parenchyma. B. Left chest ultrasonography: Presence of coalescent B lines and sub-pleural consolidations on the left posterior base.

**Figure 2:**
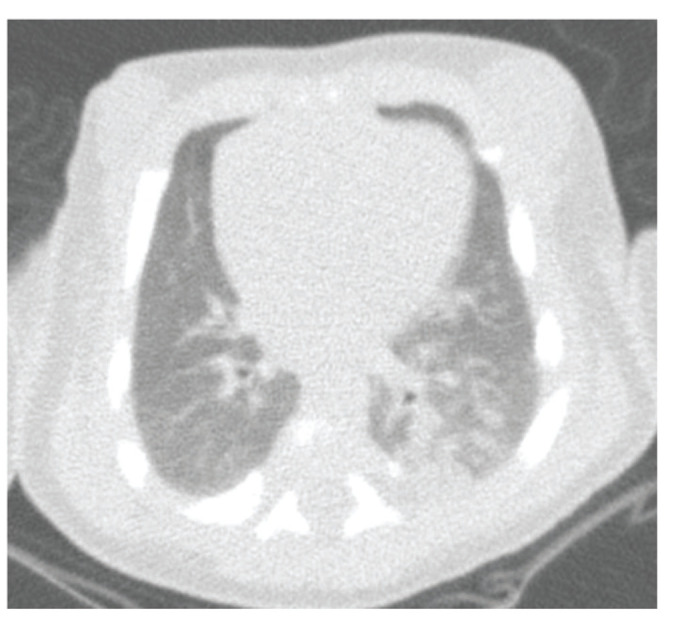
JCR chest computed tomography (case 3). Multiple pulmonary opacities and sparse focus of the consolidation of bilateral multifocal distribution, with peripheral predominance and greater extension in the left lower lobe.

**Table 1:** Clinical findings in three neonates with SARS-CoV-2 infection.

Clinical findings	Case 1-DGL	Case 2-ACO	Case 3-JCR
Age at oral swab collection (days)	2	2	8
GA (weeks)^1^	38 4/7	33 4/7	-
BW (grams)^2^	2980	2130	3600
Fever (duration)	No	No	Yes (1 day)
Respiratory distress (duration)	No	Yes (11 days)	No
Nasal congestion and runny nose	No	No	Yes
Decrease in O_2_ pulse saturation	No	Yes	No
Need for inhaled O_2_ (duration)	No	Yes (11 days)	No
Hematochezia (duration)	No	Yes (1 day)	No
Bradycardia (duration)	Yes (2 days)	No	No
Hypocalcemia (duration)	Yes (1 day)	No	No
Prematurity	No	Yes	No
Grade II intracranial hemorrhage	No	Yes	No
Anemia	No	Yes	No
Need for blood transfusion	No	Yes	No
Urinary tract infection	No	No	Yes
Length of stay (days)	8	26	14

1 Gestational age

2 birth weight.

**Table 2:** Laboratory tests in three neonates with SARS-CoV-2 infection.

Laboratory tests	Case 1-DGL	Case 2-ACO	Case 3-JCR
Hb (g/dl)	18.4	13.1	14.7
Ht (%)	48.6	35	39.8
Leukocytes (mil/mm^3^)	9520	4750	9340
Lymphocytes (mil/mm^3^)	2900	2180	6500
Neutrophils (mil/mm^3^)	5240	1770	950
C-RP (mg/dL)^1^	2.4	<0.3	5.4
APTT (sec)/R	54.6/2	11.3/0.93	-
PT (sec)/INR	10.8/0.95	50.7/1.86	-
Fibrinogen*	162	93	249
D-dimer**	2327	17559	2428

1 C-reactive protein (normal values=*200-393 mg/dL, **less than 500 ng/ml EUF [equivalent units of fibrinogen]): - not performed (the dosage was not done or we do not have the result).
